# Easy Fabrication of Highly Thermal-Stable Cellulose Nanocrystals Using Cr(NO_3_)_3_ Catalytic Hydrolysis System: A Feasibility Study from Macro- to Nano-Dimensions

**DOI:** 10.3390/ma10010042

**Published:** 2017-01-06

**Authors:** You Wei Chen, Thean Heng Tan, Hwei Voon Lee, Sharifah Bee Abd Hamid

**Affiliations:** Nanotechnology & Catalysis Research Center (NANOCAT), Institute of Postgraduate Studies, University of Malaya, Kuala Lumpur 50603, Malaysia; ashton@um.edu.my (Y.W.C.); smt8713@siswa.um.edu.my (T.H.T.); sharifahbee@um.edu.my (S.B.A.H.)

**Keywords:** cellulose hydrolysis, lignocellulosic biomass, nanocellulose, crystallinity, thermal stability

## Abstract

This study reported on the feasibility and practicability of Cr(NO_3_)_3_ hydrolysis to isolate cellulose nanocrystals (CNC_Cr(NO3)3_) from native cellulosic feedstock. The physicochemical properties of CNC_Cr(NO3)3_ were compared with nanocellulose isolated using sulfuric acid hydrolysis (CNC_H2SO4_). In optimum hydrolysis conditions, 80 °C, 1.5 h, 0.8 M Cr(NO_3_)_3_ metal salt and solid–liquid ratio of 1:30, the CNC_Cr(NO3)3_ exhibited a network-like long fibrous structure with the aspect ratio of 15.7, while the CNC_H2SO4_ showed rice-shape structure with an aspect ratio of 3.5. Additionally, Cr(NO_3_)_3_-treated CNC rendered a higher crystallinity (86.5% ± 0.3%) with high yield (83.6% ± 0.6%) as compared to the H_2_SO_4_-treated CNC (81.4% ± 0.1% and 54.7% ± 0.3%, respectively). Furthermore, better thermal stability of CNC_Cr(NO3)3_ (344 °C) compared to CNC_H2SO4_ (273 °C) rendered a high potential for nanocomposite application. This comparable effectiveness of Cr(NO_3_)_3_ metal salt provides milder hydrolysis conditions for highly selective depolymerization of cellulosic fiber into value-added cellulose nanomaterial, or useful chemicals and fuels in the future.

## 1. Introduction

For the last few decades, the energy crisis threatened the world due to the excessive utilization of the world’s depleting oil reserves by the ever-increasing human population. High worldwide demand for energy, unstable and uncertain petroleum sources, and concern over global climate change has led to the resurgence in the development of alternative renewable resources that can displace fossil fuel or petroleum-based polymers. Recently, environmental awareness has become greater and the demand for sustainable plant-based raw materials for the eco-friendly economy continues to grow into the foreseeable future [1]. In response, many countries have initiated extensive research and development programs in nanocellulose production, a green, bio-based and renewable biomaterial that has the broad possibility of use in various fields of innovative material. In fact, scholar-researchers have moved towards the utilization of this fully bio-based nanomaterial as a prominent candidate to replace synthetic reinforcing fillers in biodegradable composites and polymer matrixes as well as for the production of nanotubes and thin films [2].

Cellulose is the most abundant and renewable biopolymer available on Earth, which can be obtained from a variety of sources, including woody and non-woody plants, animals and bacteria. It has been estimated that more than 10^10^–10^11^ tons of cellulose are synthesized and destroyed globally per annum. Therefore, rational and sustainable utilization of these abundant lignocellulosic biomasses to develop new valuable bio-products would be of great benefit not only to increase renewable, value-added products but also to diminish adverse environmental/ecological impacts. Cellulose, the principal component in the plant cell wall polysaccharides, is a linear polymer built up from linearly connected β-d-anhydro-glucopyranose units (AGUs). Cellulose is a natural high-molecular-weight macro-polymer covalently linked by β-1,4-glycosidic linkages in a variety of arrangements, and several cellulose polymer chains will eventually bundle together to form fibrils or microfibrils due to its strong hydrogen bonding [3]. In nature, cellulose can be processed into its nano-dimensional structure, also known as nanocellulose via various hydrolysis treatments. From a 2015 study conducted by Usov and his group [4], it is well known that cellulose chains consist of different degrees of order, from highly crystalline arrangements to slightly perturbed distribution of chains. They proposed an alternative model to describe the ordered and disordered regions on the cellulose nanocrystals (CNC). Instead of using amorphous regions to designate the randomly packed arrangements, less-ordered surface chains may possibly be more suitable to describe the cellulose polymer chain arrangement in cellulose. In other words, the dissolution process in cellulose fibrils normally happens faster on defective crystalline parts (soft parts which are visible as kinks in single fibrils) in the long polymer chains than the crystallinity phases as there are more active sites and defect regions for chemicals or catalysts to attack. Usually, cellulose nanocrystals (CNC) can be isolated mainly by strong acid hydrolysis (e.g., 64 wt % H_2_SO_4_) in order to remove the defective crystalline domains, obtaining a typical needle-like morphology with an average diameter of 5–20 nm and fiber length up to several hundred nanometers (100–300 nm) [5]. Contrariwise, cellulose nanofibrils (CNF) are prepared by several methods, such as mechanical treatment, TEMPO-mediated oxidation, or enzymatic hydrolysis to yield long flexible fiber networks with a fibril diameter longer than CNC (5–50 nm) and length up to several micrometers, depending on its degrees of fibrillation [6]. In contrast to CNC, the produced CNF preserves the crystalline and less-ordered surface chains state in the cellulose microfibrils, rather than individual microfibrils.

Due to its nano-dimensional structure, nanocellulose possesses numerous excellent physicochemical properties, such as biodegradability, high aspect ratio, lightweight, renewability, distinctive mechanical strength with high stiffness and Young’s modulus, low density, modifiable surface properties and low coefficient of thermal expansion compared with natural cellulose [7]. Due to its high availability and remarkable properties, nanocellulose is anticipated to be as cost effective as prominent candidates to replace conventional petroleum-based polymers in various potential applications, such as bio-nanocomposites, biodegradable composites, and polymer matrixes [8]. Other potential applications of this green material include barrier films, pharmaceutical and medical applications, surface coatings, textiles and fibers, separation membrane, electroactive polymers, supercapacitors, batteries, food packaging, food additive, drug delivery, biosensors and enzyme immobilizations [9,10]. Thus, nanocellulose manufacture is currently an interesting field to study. Notably, greater length and aspect ratio CNC particles may become entangled, allowing them to reinforce composites by interacting with the polymer matrix.

In recent decades, there has been increased interest in the manufacturing of nanoscale cellulosic particles. The excitement generated is the result of its distinction properties of nanocellulose. The top-down destruction of cellulosic fibers can be conducted by mechanical disintegration [11], acid hydrolysis [12], TEMPO-mediated oxidation [13] and enzyme-assisted process [14], all of which are preferential and widely accepted as promising processes employed for converting the cellulosic feedstock to its nano-dimensional structure. Even though the mechanical process is the most direct way to produce nanocellulose by diminishing the cellulosic fibers using mechanical stress along the longitudinal axis of the cellulose structures, the treatment is not cost effective due to high energy consumption, the involvement of complex equipment, and repetitive process. In addition, enzymes hydrolysis is a costly treatment as the enzymes are hard to recycle and the process requires a longer period (2–6 weeks) to achieve a satisfactory conversion (~80%) [15]. Therefore, acid hydrolysis conducted by strong mineral acid such as sulfuric (H_2_SO_4_), hydrochloric (HCl), and phosphoric acid (H_3_PO_4_) is recognized as the most effective to hydrolyze defective cellulose crystalline parts while leaving the highly crystalline nano-sized cellulose segments unaltered [16]. In the case of strong acid treatment, the hydronium ions (H_3_O^+^) will attack and cleave β-1,4-glycosidic linkages within the single cellulose chains as well as break down the extensive network of intra- and intermolecular bonds between the cellulose chains [7,17]. Unfortunately, the yielded sulfated nanocellulose suffers from low product yield and less thermal stability compared to that of its starting material due to the introduction of active sulfate ester groups on the fiber surface that can be detrimental to the thermal stability of treated product [18]. Moreover, this process is highly corrosive, hazardous, and requires harsh conditions and the involvement of tedious neutralization treatment of concentrated acidic effluent, which further limits its industrialization. For HCl hydrolysis, the aqueous nanomaterial suspensions tend to flocculate rather than well-disperse, and only a low yield of 20% could be obtained [19]. Even if some studies reported a higher yield of 60.5% via phosphotungstic acid hydrolysis, the hydrolysis efficiency was low and the reaction process is time-consuming [20], or else mechanical activation (ultrasonication, ball milling or mechanochemical) is necessary to enhance the competence [21]. Similar to that of diluted or organic acids that have been proposed for milder hydrolysis reactions, such treatments were less effective in hydrolyzing the high recalcitrant cellulose macromolecule. For these reasons, an increasing effort has been made to use the other mineral acids for cellulose hydrolysis. Recently, the mixed acid solution (hydrochloric acid and sulfuric acid) may be a good choice for the production of CNC suspension, but this process still also took a long period.

According to some published papers, the typical hydrolysis methods, such as TEMPO-mediated oxidation and mechanical disintegration are popular in the preparation of CNF from various cellulosic feedstocks. Briefly, TEMPO process is initiated by (2,2,6,6-tetramethylpiperidin-1-yl)oxidanyl, and the CNF obtained are more uniform and can be well dispersed in aqueous solution. In contrast, mechanically induced deconstructing strategies by different approaches (such as grinding, microfluidization, high-pressure homogenization, high-speed blending, refining, cryocrushing or ultrasonication) are considered as promising methods for isolating CNF. However, large-scale applications of these techniques have several restrictions. In fact, mechanical refining could efficiently separate the microfibrils, but the high crystalline ordered structure might be broken due to the lower selectivity of high shearing force, resulting in a lower crystallinity. Likewise, the TEMPO-oxidation process may turn some of the crystalline cellulose molecules into disordered structures during the oxidation and the resultant CNF product exhibited a lower CrI value, even compared to its starting material [22]. Ultrasonication is another renowned way for CNF fabrication in which 20–50 kHz of ultrasound is applied to defibrillate cellulose for cell disruption. This is a greener hydrolysis technique as no chemical is involved during the generation of nanocellulose. The high frequency is generated and converted into the mechanical energy, which can be transmitted to cellulose via a metal probe. Unfortunately, the product obtained by this technique is a mixture of nano- and microfibers since some of the fibrils tended to be peeled off from the original cellulose fibers while some still remain on the surface of fibers [23]. In addition, cellulose can be cycled through a high-pressure homogenizer to yield nanofibers. Typically, smaller and more uniform nanoparticles can be obtained with increasing number of homogenization cycles, but such high-pressure treatment is more likely to reduce and/or destroy the crystalline domains by separating the cellulose molecular mass, or instead fail to defibrillate the cellulosic pulp sufficiently [24]. It is worth mentioning that the energy demand increases significantly with the increase of homogenizing time, and this may be the main drawback of application for CNF. The main issue often experienced by a homogenizer is extensive clogging and flocculation of the nozzle as the CNF tend to aggregate, and this means that extra capital cost is required to overcome this frequent issue. Therefore, some authors proposed that an additional pre- or post-treatment is necessary in order to obtain nanofibers product, such as hydrolysis, grinding, homogenization, or other mechanical treatments. 

To accomplish a technical feasibility and high selectivity controllable hydrolysis pathway, transition metal salt catalysts have been used as a potential hydrolyzing agent in the cellulose depolymerization process. Today, metal salts have been discovered to have the following notable advantages over inorganic acids or organic solvents in cellulose hydrolysis: (i) can disrupt the hydrogen bonds more efficiently and induce the degradation of cellulose; (ii) less corrosive and more environment-friendly; and (iii) better contact between molten state metal salts and solid cellulose [25]. According to literature studies, chromium-based metal salt catalysts have been found to be highly effective in direct conversion of cellulosic resources into different chemicals and liquid fuels such as glucose, hydroxymethylfurfural (HMF), xylose and levulinic acid. Binder and Raines [26] reported that CrCl_2_-catalyzed hydrolysis of cellulose could produce a high yield of HMF (ca. 54%) in the N,N-dimethyl acetamide solvent containing [EMIM]Cl and lithium chloride (LiCl) additives at 140 °C for 2 h. Su et al. [27] investigated the effect of CrCl_2_ in [EMIM]Cl ionic liquid on the cellulose hydrolysis, and the results revealed that 58% of HMF was successfully achieved under the reaction conditions of 120 °C for 8 h. In recent years, a study performed by Peng et al. [28] found that CrCl_3_ catalyst was exceptionally effective in the conversion of cellulose to levulinic acid with a maximum yield of 67 mol % at 200 °C for 180 min. During the catalytic hydrolysis, the multivalent metal ions act as a Lewis acid that possess a high ability for hydrolyzing the cellulose matrix by disrupting the bonding system to produce fermentable sugar molecules, and it had better efficiency compared to diluted acid alone [29]. Therefore, it is believed that the intermediate solid crystalline nanocellulose could be produced instead of water-soluble monomer molecules under controlled low severity hydrolysis settings.

In this study, we propose for the first time the use of Cr(NO_3_)_3_ hydrolysis system as an efficient and sustainable technique for producing nanocellulose. Many papers have reported the combination of metal salts and mineral salt or mechanical process for hydrolyzing the cellulosic feedstock into hydrocellulose or nanocellulose, but there is limited research on the involvement of metal ion catalyst alone to produce cellulose nanocrystals (CNC) without the addition of any mineral acid or assistance of mechanical treatment. The main objectives of this study include the following: (i) exploiting the feasibility and practicability of Cr(NO_3_)_3_ as a high potential catalyst for converting cellulose into its nanostructured morphology; (ii) optimizing the reaction conditions of Cr(NO_3_)_3_ hydrolysis system in order to produce a high performance nanocellulose; and (iii) evaluating the physicochemical changes in terms of functional groups (Fourier transform infrared; FTIR), crystalline structure (X-ray diffraction; XRD), morphological properties (Field emission scanning electron microscopy; FESEM and transmission electron microscopy; TEM) and thermal stability (Thermogravimetric analysis; TGA) during the production of nanocellulose from macro-sized native cellulose. We also attempted to produce CNC from the same starting material by concentrated H_2_SO_4_ hydrolysis, in order to perform a comprehensive comparison and assessment between resulting nanocellulose (CNC_Cr(NO3)3_ and CNC_H2SO4_) using a similar analytic instrument systematically. We used microcrystalline cellulose (MCC) as a starting material instead of extracted cellulosic pulp from lignocellulosic biomass to better understand the role of Cr(NO_3_)_3_ hydrolysis system in the conversion of cellulose, avoiding impure bulk materials that might cause irreproducible results.

## 2. Results and Discussion

### 2.1. Effect of Reaction Temperature

**Figure 1:**
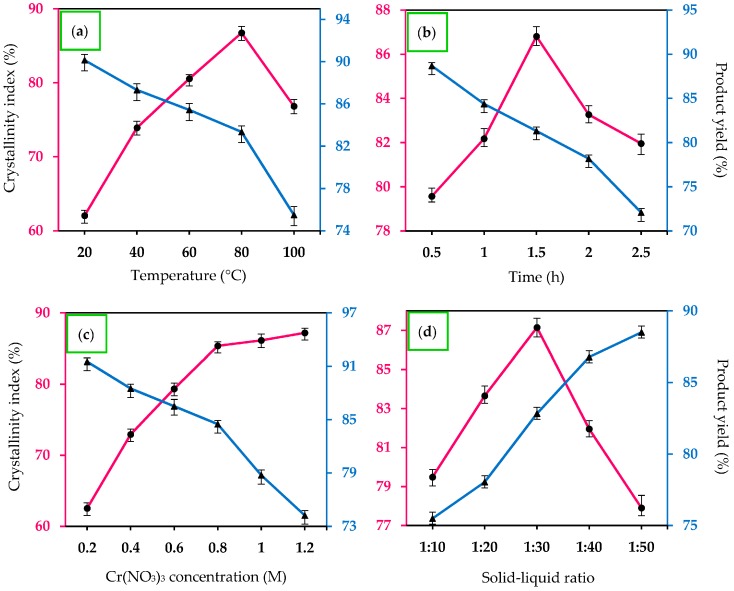
Effects of: (**a**) Reaction temperature; (**b**) Hydrolysis time; (**c**) Cr(NO_3_)_3_ concentration; and (**d**) Water content on the crystallinity index and yield of nanocellulose.

Reaction temperature plays an important role in initiating the hydrolytic depolymerization process of cellulose and eventually enhancing the chemical degradation of cellulose macromolecule into the nano-dimensional. The crystallinity index of yielded nanocellulose affected by reaction temperature is shown in Figure 1a. The reaction temperature was set at different levels (20, 40, 60, 80 and 100 °C) while other operating parameters were fixed, including Cr(NO_3_)_3_ concentration (0.8 M), reaction time (1.5 h) and solid–liquid ratio (1:30). The crystallinity index of nanocellulose was significantly increased (62.1% to 86.7%) with the rise of temperature from 20 to 80 °C. The increment was mainly contributed by the increased diffusion rate of H_3_O^+^ and Cr^3+^ ions in aqueous solution and penetration into loosely-packed non-crystalline regions of cellulose to cleave the glycosidic bonds. In fact, the hydrolysis reaction is generally more kinetically favorable, which means that a higher reaction temperature could significantly enhance the degree of progressive removal of defective crystalline domains in the cellulose matrix, releasing the individual crystallite segments [7]. This led to the breakage of the glycosidic linkage in cellulose long polymeric chains into a smaller dimension. However, the extremely high temperature of 100 °C resulted in the destruction of the crystalline structure of cellulose under excessive heat energy, and eventually promoted the formation of unfavorable side products, such as levulinic acid and HMF [30]. Therefore, the optimal reaction temperature of 80 °C was selected to conduct further experiments with the advantages of energy-saving, ability to produce higher crystallinity product and, most importantly, the reaction was more manageable under normal conditions of atmospheric pressure.

Surprisingly, the yield of produced CNC_Cr(NO3)3_ gradually decreased with increasing hydrolysis temperature, which decreased to ca. 75% when the reaction temperature rose from 20 to 100 °C. This finding suggested that less-ordered cellulosic surface chains started to disintegrate into water soluble product by the acidic solution, which in turn lowered the final yield of the nanomaterial [31]. It was observed that the yield of nanocellulose dropped sharply from 83.1% to 75.4% when the reaction was heated up from 80 to 100 °C. Therefore, the reaction parameters must be well-controlled in order to compromise between product yield and the crystallinity index of the nanocellulose particles.

### 2.2. Effects of Hydrolysis Time

Besides the reaction temperature, hydrolysis time also presents a significant effect on the crystallinity index of nanocellulose, which is illustrated in Figure 1b when the hydrolytic depolymerization of cellulose was conducted at 80 °C, 0.8 M Cr(NO_3_)_3_ concentration and solid–liquid ratio of 1:30. The nanocellulose yield showed a gradual decline trend while the crystallinity index of nanocellulose was increased over time. The increment reached a maximum of 86.8%, followed by a decreasing trend when the reaction was further prolonged to 2.5 h. The decrement was due to unfavorable hydrolysis process occurring in the crystalline cellulose, which led to the damage of high crystalline segments. However, insufficient reaction time (0.5 h) resulted in incomplete hydrolysis process of breaking down the strong bonding network of cellulose macromolecules, contributing to a low crystallinity index (79.6%). It is widely accepted that increasing contact time between hydrolyzing catalyst and reactant would eventually cause the swollen cellulose and even the enlargement of the intra- and inter-fiber pores of treated matrix, so that the degradation of cellulose network structure can happen effectively. This would also allow the catalyst to penetrate into the interior part of the cellulose pulp to liberate the nanoscale cellulose segments to accelerate the production of nanocellulose. For these reasons, it is very important to choose an appropriate hydrolysis time to maximize the removal of non-crystalline areas in cellulose, while preserving the crystalline parts. Therefore, hydrolysis time of 1.5 h was chosen as the optimal preparation condition and was used in the subsequent experiments, as the extension of time would cause a negative effect in enhancing the crystallinity of nanocellulose.

### 2.3. Effects of Metal Salt Concentration

Theoretically, higher catalyst concentration can enhance the degree of hydrolysis of cellulose. Unfortunately, the hydrolysis process is not only occurs in the cellulose defect domains but also in its crystalline structure. The defective crystalline phase in cellulose chains is more easily decomposed to its water soluble oligomers in the presence of metal ion catalyst than that of crystalline phase owing to its tightly packed structure and strong hydrogen bonding [32]. Thus, enhancing the hydrolysis degree of less-ordered cellulose phase by increasing the metal ion concentration benefits the production of high crystallinity nanocellulose. However, it is necessary to find an optimal metal ion concentration, as it is possible that some crystalline nanocellulose segments could gradually be hydrolyzed to further convert to liquid organic molecules as the secondary product when treated with an extremely high concentration of metal salt catalyst. Figure 1c reveals that when Cr(NO_3_)_3_ concentration increased from 0.2 to 1.2 M, the nanocellulose crystallinity progressively increased from 62.5% to 87.1% while maintaining reaction duration for 1.5 h, and a solid–liquid ratio of 1:30 at 80 °C. Simultaneously, the product yield decreased almost linearly with increasing concentration of metal salt. This implied that the successive degradation of defective crystalline allomorphs after the catalytic hydrolysis process induces the exposure of high crystalline segment in the nanocellulose, which leads to increasing crystallinity. However, it is worth noting that the increasing tendency of crystallinity index became slower when the metal salt concentration was higher than 0.8 M. Therefore, for better economic competence, 0.8 M metal salt catalyst was selected as the optimum concentration for hydrolysis.

In this study, Cr(NO_3_)_3_ acted as a Lewis acid to react with cellulose molecules for the production of the nanostructured product. The possible mechanism behind this phenomenon is that Cr^3+^ ions can dissociate into a coordination complex structure with water molecules (H_2_O) at the initial hydrolysis stage. The H_2_O molecules tend to be polarized by this central metal ion by withdrawing its electron density, resulting in the H-atoms in hydroxyl groups (O–H) becoming more electropositive. However, the original state of these complex ions was unstable and there was a high probability of becoming deprotonated by releasing hydronium (H_3_O^+^), further enhancing the acidity of the reaction mixture [33]. The detail reaction steps are as follows:

Cr(NO_3_)_3_ + 6H_2_O → [Cr(H_2_O)_6_]^3+^ + 3NO_3_^−^ (1)

[Cr(H_2_O)_6_]^3+^ + H_2_O → [Cr(H_2_O)_5_(OH)]^2+^ + H_3_O^+^ (2)

[Cr(H_2_O)_5_(OH)]^2+^ + H_2_O → [Cr(H_2_O)_4_(OH)_2_]^+^ + H_3_O^+^ (3)

[Cr(H_2_O)_4_(OH)^2^]^+^ + H_2_O → [Cr(H_2_O)_3_(OH)_3_] + H_3_O^+^ (4)

Enhancing the acidity of the hydrolytic system would weaken and thus promote the breakdown of anhydro-glucopyranose units between cellulose polymeric chains, producing smaller cellulose units. Otherwise, there were insufficient hydronium ions present in the reaction system if the metal salt concentration was low. In addition, the Cr^3+^ metal ions could easily interact with the oxygen atoms of C–O–C glycosidic bonds between the glucose units, leading to the formation of the oxygen-chromium complex intermediate. Due to the adsorbed metal ions, the bonding energy of oxygen atoms and carbon atoms of pyranose rings in the intermediate were reduced because of the increase of its bond length and bond angle [29]. Therefore, the activation energy of the hydrolysis process was reduced. However, as stated earlier, the operational conditions of the hydrolysis reaction must be optimized in order to prevent the carbonization process (formation of char).

### 2.4. Effects of Water Content

As shown in Equations (1)–(4), water molecules play a vital role in initiating the Cr^3+^-catalyzed hydrolysis process by generating H_3_O^+^ ions. In order to accelerate the rate of the hydrolysis reaction, it was suggested that water content be increased in order to produce more hydronium ions by shifting the reaction forward to the product side (Le Chatelier’s principle). Therefore, the effect of solid-liquid ratio of crystallinity index of nanocellulose was investigated by varying the ratio from 1:10 to 1:50 at a constant metal salt concentration (0.8 M), and 1.5 h reaction at 80 °C. As shown in Figure 1d, the nanocellulose yield increase when more water was added into the hydrolysis system (higher solid-liquid ratio); this might be similar to decreasing the metal salt concentration. In other words, the acidity of the reaction mixture was reduced by water molecules. However, the crystallinity of nanocellulose reached a maximum value (87.2%) at the solid-liquid ratio of 1:30 and gradually decreased when the cellulose matrix was treated with a more diluted hydrolysis system (1:40 and 1:50). This result might reflect that a small amount of H_2_O could reduce the cellulose hydrolysis efficiency due to the incompletion reaction between the cellulosic material and hydrolyzing catalyst. In addition, it was observed that some portion of the cellulosic material failed to immerse completely in the reaction solution when the solid-liquid ratio set at 1:10, which also leads to the hydrolysis process not taking place effectively. However, the catalyst concentration significantly reduced when a large amount of water was introduced to the reacting system, and this eventually led to a decrement of the crystallinity of nanocellulose as shown in Figure 1c. In fact, it is important to control the consumption of water in actual industrial production. Taking the cost and efficiency into consideration, 1:30 was chosen as a suitable loading ratio between cellulose solid material and the catalyst aqueous solution.

From the several single-factor experiments conducted, it can be summarized that the optimum operational conditions for cellulose hydrolysis were determined as 1.0 g of cellulose material and 0.8 M of Cr(NO_3_)_3_ catalyst with a solid-liquid ratio of 1:30 at the temperature of 80 °C for 1.5 h. In addition, the yield of solid product cellulose nanomaterial (CNC_Cr(NO3)3_) was approximately 83.6% ± 0.6%. Meanwhile, the CNC_H2SO4_ obtained via sulfuric acid hydrolysis was determined to produce about 54.7% ± 0.3%. The yield of CNC_H2SO4_ was much lower than that of CNC_Cr(NO3)3_ as an acid hydrolysis process is well-known to be a harsh treatment that results in disintegration and degradation, leading to fewer crystalline domains, causing the dramatic decrease in product yield. Both manufactured CNC products were further characterized.

### 2.5. Crystallinity Study—XRD Investigation

**Figure 2:**
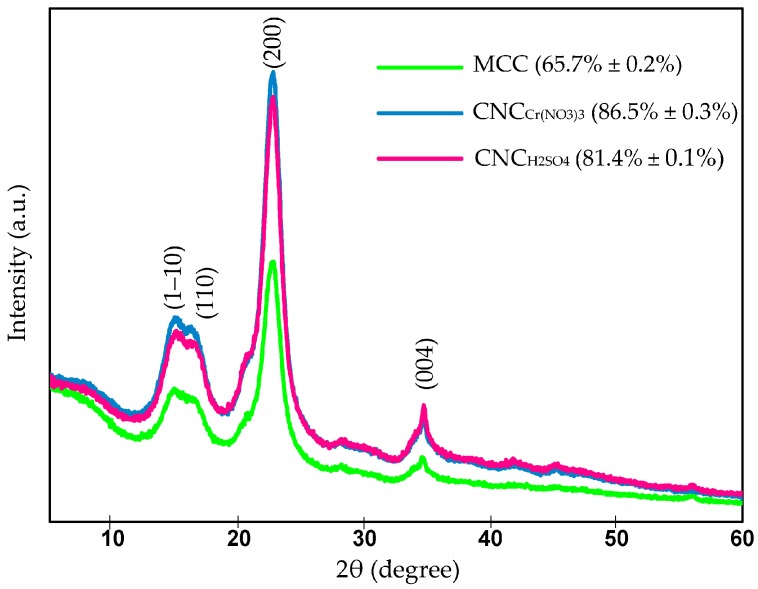
X-ray diffraction (XRD) patterns of the native cellulose, CNC_H2SO4_ and CNC_Cr(NO3)3_. The parenthesis refers to the crystallinity index of the samples.

It is widely recognized that natural state cellulose consists of both less-ordered and highly-arranged crystalline regions in their molecular structure, and wide-angle X-ray scattering (WAXS) analysis was conducted to determine the crystalline index of native cellulose and optimized nanocellulose. XRD studies were performed to evaluate the crystallinity behaviors of native cellulose and yielded nanocellulose specimens. As shown in Figure 2, the XRD diffractogram profiles for all samples exhibited a similar pattern, which represented the typical semi-crystalline materials with a crystalline peak and non-crystalline broad halo. In addition, all XRD profiles showed the major peaks at around 2θ = 15.1 (1–10), 16.5 (110), 22.5 (200), and 34.6° (004), which indicated the presence of cellulose I structure [17,34]. From the X-ray curves, it was clearly observed that the XRD patterns of both nanocellulose samples and commercial MCC were similar. This observation suggested that the crystalline structure of cellulose I of MCC had been well maintained after the hydrolysis process to different extents.

The crystallinity index for native cellulose, CNC_H2SO4_ and CNC_Cr(NO3)3_ were calculated based on Equation (5) and found to be 65.7%, 81.4% and 86.5%, respectively. It was noticeable that the produced CN_CH2SO4_ and CNC_Cr(NO3)3_ exhibited higher crystallinity compared with starting material. This is because of the successive removal of the less-ordered surface cellulose chains in the matrix during the catalytic hydrolysis process, leading to the exposure of solid elementary crystalline cellulose phases [35]. In this study, the untreated MCC was less crystalline than the treated ones. The higher intensity peak associated with CNC_H2SO4_ and CNC_Cr(NO3)3_ was predominantly due to the successive dissolution of the defective cellulose regions with the consequent increase in the sample crystallinity index. Although H_2_SO_4_ and Cr(NO_3_)_3_ exhibited the strong catalytic effects toward the degradation of cellulose, the maximum crystallinity index was obtained with CNC_Cr(NO3)3_ (86.5% ± 0.3%) compared to that of CNC_H2SO4_ (81.4% ± 0.1%). In the process of hydrolysis, the H_3_O^+^ ions tend to penetrate into the non-crystalline regions of the cellulose matrix, promoting the hydrolytic cleavage of glycosidic linkages and eventually releasing the individual crystallites. On the other hand, the higher crystallinity of nanocellulose product could be attained because the nanocellulose tends to be realigned into a better organized and crystalline structure, which results in the self-assembly phenomenon of nanocellulose, enabling close packing and hydrogen bond formation [36]. Besides that, there is no remarkable difference in the XRD patterns between CNC_H2SO4_ and CNC_Cr(NO3)3_. These results confirmed that the proposed Cr(NO_3_)_3_ hydrolysis system does not have a negative effect on the crystallites structure of cellulose. 

In this study, the crystallinity index of CNC_H2SO4_ was lower than that of CNC_Cr(NO3)3_, which could be ascribed to the reasonable explanation that the strong H_2_SO_4_ not only attacks the randomly-ordered structure in cellulose but also destroys the crystalline one during hydrolysis. For industrial uses, the addition of nanocellulose to a polymer matrix is one of the effective ways to reduce the oxygen transmission rate in packaging application; however, the slightly lower crystallinity of CNC_H2SO4_ might have a negative effect on its gas barrier property [22,37]. 

### 2.6. Chemical Structures—FTIR Analysis

**Figure 3:**
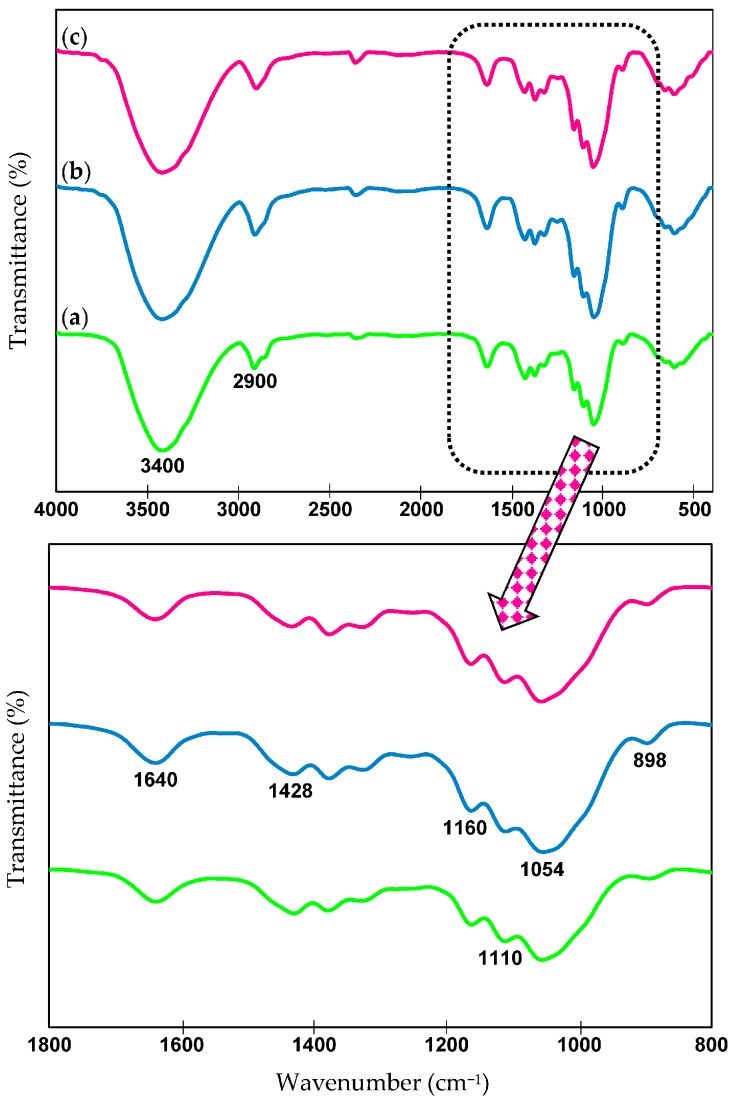
Fourier Transform Infrared (FTIR) spectra of: (**a**) Native cellulose; and CNC isolated via (**b**) H_2_SO_4_; and (**c**) Cr(NO_3_)_3_ catalyst.

FTIR spectroscopy is an appropriate technique to evaluate the changes in the chemical compositions of the samples in response to native cellulose and yielded nanocellulose (CNC_H2SO4_ and CNC_Cr(NO3)3_). As presented in Figure 3, the FTIR spectra of all the samples exhibited two major absorbance regions, which included the high (3500–2800 cm^−1^) and low wavenumbers (1700–500 cm^−1^) regions, consistent with previous studies [38,39]. A broad and dominant absorbance peak located in the region of 3600 to 3200 cm^−1^ was observed in all spectra, which corresponded to the characteristic of hydrogen bonding O–H stretching vibrations [40]. In addition, C–H stretching gave rise to the band at 2900 cm^−1^ [21]. A small peak was detected at 1640 cm^−1^ associated with –OH bending vibration of absorbed water by the fibers as the cellulose is hygroscopic in nature [41], whereas the peak at 1430 cm^−1^ related to the intermolecular hydrogen attraction at the C_6_ group [7]. Although all the samples were subjected to a proper drying process prior to FTIR analysis, the complete elimination of moisture in the samples was very difficult due to the strong cellulose–water interaction [42]. Moreover, the stretching vibration of the C–O–C pyranose ring within the cellulose molecules was observed at a sharp peak of 1054 cm^−1^ [43]. Besides that, the fingerprint region of the FTIR spectra, which lies in the range within 600 to 1100 cm^−1^, is a significant signal for tracking the presence of β-glycosidic linkages that contributed by the vibration of wagging, deformation and twisting modes of the anhydro-glucopyranose units [5]. Furthermore, the absence of NO_3_^−^ peaks at 1384 cm^−1^ in the spectra implied the complete removal of Cr^3+^ metal salt during the washing by centrifuge and dialysis process [17].

Based on the FTIR spectra obtained from native cellulose and yielded nanocellulose, there were no significant changes in the functional groups after the cellulose hydrolyzed by H_2_SO_4_ and Cr(NO_3_)_3_ hydrolyzing catalysts, except peak intensities. Therefore, this suggested that the chemical structure of yielded nanocellulose that was not altered after the hydrolysis process, where the typical structure of the parent cellulose remained well-preserved, only different in term of morphology and crystallinity. This observation was in good agreement with other works of literature [7,17,21,44] exhibited a similar FTIR pattern compared to its corresponding cellulosic materials. It is important to note that the extracted nanocellulose exhibited absorbance signals at 1428, 1160, 1110, and 898 cm^−1^, suggesting that nanocellulose was primarily in cellulose I structure [45].

### 2.7. Morphological Investigation—FESEM-EDX

**Figure 4:**
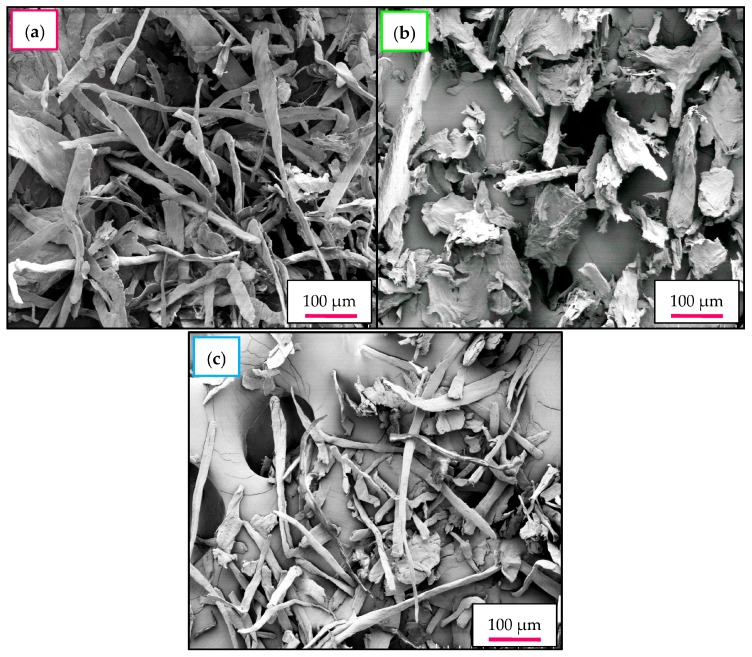
FESEM images of: (**a**) Native cellulose; (**b**) CNC_H2SO4_; and (**c**) CNC_Cr(NO3)3_. It is worth noting that the cellulose fiber is diameter greatly reduced after hydrolysis.

Figure 4a illustrates that native cellulose is primarily comprised of aggregated cellulosic fibrils with an irregular shape. In nature, each cellulose fiber is made up of several to hundreds of microfibers that tend to be assembled together and lead to the formation of the compact structure of cellulose [7]. The aggregation of microfibers can contribute to the strong hydrogen bonding between the individual cellulose chains [46]. In addition, each elementary cellulose fiber appeared to be long in length with a rough surface and ow aspect ratio. The aggregation of native cellulose is due to the strong hydrogen bonding system between each individual cellulose microfibril in the macrostructure of cellulose, which is consistent with the literature [7].

The long cellulosic fibrils were broken down to a great extent after the hydrolysis treatment catalyzed by Cr(NO_3_)_3_ and H_2_SO_4_, which can be clearly observed in the FESEM micrographs as presented in Figure 4b,c. Smaller and more individualized fragments were successfully disintegrated and/or separated from the bundles of micro-sized cellulose fibers, which resulted in further reduction of its diameter. These findings were in excellent accordance with the reported literature [7]. The intermittent breakdown in the fibrillar structure of CNC in this study could be correlated with the successive hydrolysis treatment initiated by the Cr(NO_3_)_3_ metal salt and H_2_SO_4_ in the dissolution of the less-ordered defective crystalline regions as well as the hydrolytic cleavage of β-1,4-glycosidic linkages. The highly ordered crystalline arrangements appear in nanocellulose samples due to the formation of intra- and intermolecular H-bonding between the hydroxyl groups.

In the present study, finer and shorter nanoscale fibrils were expected in the CNC_H2SO4_ and CNC_Cr(NO3)3_; however, this was not obvious from the FESEM micrographs, as shown in Figure 4b,c. This was accredited to the strong intermolecular hydrogen bonding within the cellulose chains. As a result, the cellulose fibrils tended to agglomerate with each other during the freeze-drying process owing to the higher number of OH groups on the cellulose fibrils. A similar morphology has also been reported for the nanocellulose derived from flax and microcrystalline cellulose [47]. In order to confirm the successive production of nanocellulose, more insight into the particle size and dimension of CNC_H2SO4_ and CNC_Cr(NO3)3_ in suspension was observed through TEM analysis.

The results of EDX analyses of the CNC_H2SO4_ and CNC_Cr(NO3)3_ are given in Table 1. The chemical analysis of both nanocellulose products revealed carbon (C) and oxygen (O) as its major elements. However, the elemental analysis by EDX identified that a small amount of sulfur (S) content was introduced on CNC specimen treated by H_2_SO_4_. The trace amounts of sulfur components probably contributed by the sulfate groups that came from H_2_SO_4_. In fact, for sulfuric acid hydrolyzed CNC, sulfur will always be present since the sulfate half esters have a role in stabilizing the CNC aqueous suspensions and are introduced during the hydrolysis step. Moreover, the presence of sulfur indicated that the chemical interaction of sulfuric acid with the surface of cellulose fiber, and, most importantly, this also further evidenced that cellulose fiber had been hydrolyzed by H_2_SO_4_ during the treatment. Sulfated nanocellulose was believed to be due to the esterification process occurred during the acid hydrolysis according to the procedure as follows [7]:

Cellulose–OH + HOSO_3_H → Cellulose–OSO_3_H + H_2_O

Most importantly, the elemental chromium was not detected in CNC_Cr(NO3)3_, which indicated that Cr^3+^ could be completely removed during centrifugation steps. Thus, it can be concluded that the yielded CNC_Cr(NO3)3_ had been washed cleanly after the hydrolysis process and metal ion was completely detached in the treated samples. This finding was in good agreement with FTIR result.

### 2.8. Structural Morphology and Particle Size Distribution—TEM Analysis 

**Figure 5:**
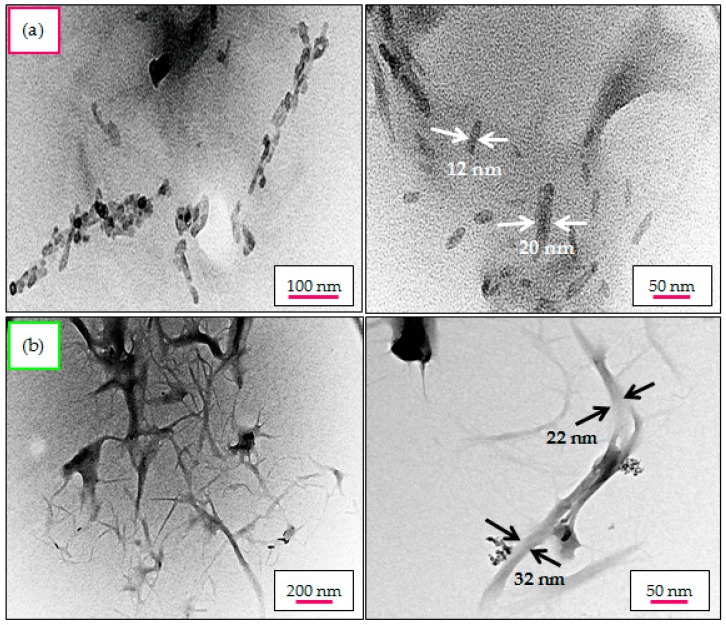
TEM micrographs of nanocellulose treated by: (**a**) H_2_SO_4_; and (**b**) Cr(NO_3_)_3_ catalyst, highlighting the different morphologies observed in both different cellulose nanomaterials (**right** image has a larger magnification than **left** image).

Because the manufactured nanocellulose products were matted and not individualized as seen in FESEM images, the nanoparticles size and morphology of CNC_H2SO4_ and CNC_Cr(NO3)3_ were confirmed by transmission electron microscopy (TEM). This technique is considered one of the most accurate methods for direct measurement and observation at a nanoscopic scale. Under the controlled hydrolysis conditions, the non-crystalline regions of cellulose were cleaved transversely by H_2_SO_4_ and Cr(NO_3_)_3_ hydrolyzing catalysts with heat treatment. The average diameter of the nanocellulose fibers was calculated by ImageJ software for at least 200 measurements. The results showed that the macro-sized native cellulose fiber diminished to the nanometer scale after the hydrolysis reaction. However, it can be clearly observed that the morphology of the nanocellulose obtained for both hydrolysis procedures exhibited noticeably different structures: CNC_H2SO4_ displayed a rice-shape structure (Figure 5a) and CNC_Cr(NO3)3_ revealed a spider-web-network-like structure (Figure 5b), confirming a successful extraction of nanocellulose from MCC. However, part of the CNC_H2SO4_ and CNC_Cr(NO3)3_ samples were observed in a somewhat agglomerated form, as bundles of particles with some dispersed individual fibrils present. In fact, it was challenging to obtain separated nanocellulose fibrils due to the presence of strong hydrogen bonding and the high surface area between these fibrils fosters agglomeration, overlapping and assembly. This phenomenon occurred mainly during the evaporation of the dispersing medium in order to dry the particles for TEM imaging [48]. Similar observation has been previously reported [49].

Besides the morphological aspects, the TEM images allowed the observation of a variation in the average particle width (d) and length (L), thus enabling determination of the aspect ratio (L/d) of the yielded nanocellulose products. Significant differences were noticed for the average particle length. It was clearly observed that the average diameter (d) of CNC_Cr(NO3)3_ was comparable to that of CNC_H2SO4_, the length (L) of the former product seems to be much longer than the latter product, and this gave rise to the presumably higher aspect ratio (L/d). The use of MCC as starting material hydrolyzed in the H_2_SO_4_ system led to the formation of CNC_H2SO4_ having an average fiber width of approximately 9.9 ± 3.2 nm with an average length of 34.8 ± 14.2 nm providing an aspect ratio of 3.5. Meanwhile, the inorganic salt-treated CNC_Cr(NO3)3_ displayed a mean width of 29.1 ± 7.8 nm, a length of 455.7 ± 38.1 nm and an aspect ratio of 15.7. Briefly, CNC_Cr(NO3)3_ rendered stronger reinforcing effect (i.e., higher tensile strength and modulus) than that of CNC_H2SO4_ contributed by its high aspect ratio, making it become an ideal reinforcing agent for polymer matrix [50]. In addition, CNC_Cr(NO3)3_ with higher aspect ratio might exhibit higher optical transmittance when it is used for preparing nano-paper or reinforcement for polymer composites [37].

In this study, the aspect ratio values of CNC_Cr(NO3)3_ are well above 10 (~15.7), which is considered the minimum value required for a good stress transfer from the matrix to the fiber for a good reinforcement when nano-biocomposite is targeted [51]. Generally, it is known that to constitute the composites, nanocellulose structures with a higher aspect ratio have a good reinforcing capability of the final polymer products, resulting in an improvement in the thermal and mechanical properties [52]. Besides the outstanding mechanical properties, another advantage of Cr(NO_3_)_3_-catalyzed hydrolysis over H_2_SO_4_ hydrolysis is that no sulfate groups were introduced to the surface of cellulose fibrils after the hydrolysis process. 

### 2.9. Thermostability Analysis 

**Figure 6:**
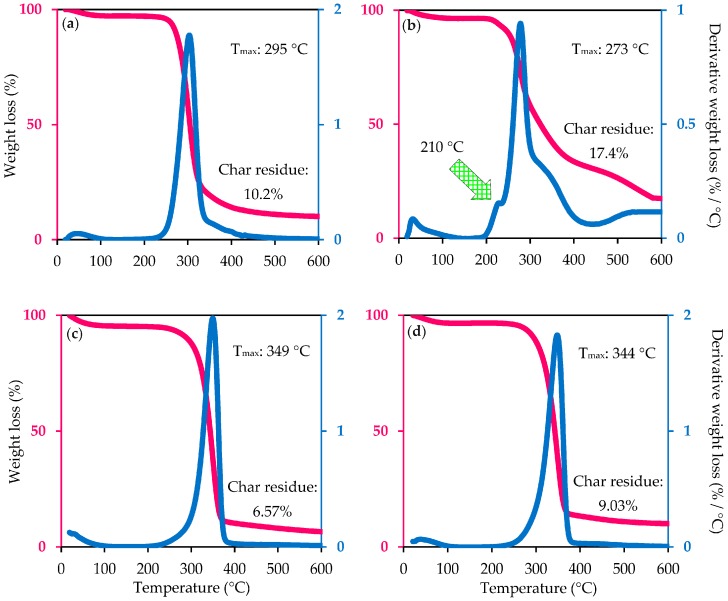
Thermogravimetric (TG) and its derivative curves (DTG) of the: (**a**) Native cellulose; (**b**) Sulfated CNC_H2SO4_; (**c**) Neutralized CNC_H2SO4_; and (**d**) CNC_Cr(NO3)3_.

The pyrolytic study was used to investigate the thermal stability of native cellulose, CNC_H2SO4_, and CNC_Cr(NO3)3_. Determination of thermal properties of reinforcing materials is an important parameter in order to evaluate and identify their applicability of these cellulose nanomaterials in biocomposite applications which are always processed at high temperatures. Both TG and DTG curves were plotted and graphically presented in Figure 6 to track the thermal stability differences between native cellulose and yielded nanocellulose samples (CNC_H2SO4_ and CNC_Cr(NO3)3_). In fact, DTG curves provided a more precise assessment and comparison, especially in term of maximum decomposition temperature (T_max_) value.

An initial weight loss was observed for all cellulosic samples in the region below 100 °C, mainly corresponding to the evaporation of moisture content or any other volatile low-molecular-weight compounds loosely bound to the surface or inside the materials [42]. The presence of absorbed water was also observed by FTIR characteristic peaks at the signal of 1640 cm^−1^, which represents the bending vibration of intermolecular hydrogen bonding of water molecules. The DTG curve revealed there is a major difference in the decomposition temperature between native cellulose and CNC_H2SO4_, in which the CNC_H2SO4_ started to undergo a degradation process much earlier (at 210 °C) than that of cellulose as well as CNC_Cr(NO3)3_. This phenomenon was accredited to several possible reasons:

(i) The replacement of hydroxyl groups (O–H) by active sulfate groups (O–SO_3_H) via either esterification or direct catalysis would initiate the dehydration reaction to occur on the sulfated nanocellulose fiber, leading to the water formation, further catalyzing the decomposition of remaining cellulose fiber into smaller particles. On the other hand, the unsulfated crystals tend to collapse at higher decomposition temperature [53];
(ii) It was believed that the concentrated H_2_SO_4_ not only removes the loosely-packed defective parts but also has high potential to damage crystalline domains, making the molecules more susceptible to the degradation step in response to increased temperature [18];
(iii) The rapid reduction of molecular weight of nanocellulose during hydrolysis compared to that of its starting material contributed to its early decomposition during heating treatment [54];
(iv) The short nanocellulose chains provide a high specific surface area and result in the formation of a large number of free-end-chains in the surface that tended to decompose easily at a lower temperature. The high surface area of nanocellulose plays a significant role in diminishing their thermal stability due to the increased exposure to heat source [55].

Thermal degradation of native cellulose started at approximately 225.4 °C in an N_2_ atmosphere, while the CNC_Cr(NO3)3_ product degradation began at ca. 238.2 °C. The increased thermal stability of CNC_Cr(NO3)3_ might be attributed to the removal of defective crystalline cellulose, as evidenced above by XRD results. Nevertheless, the CNC_H2SO4_ showed a lower thermal stability (201.2 °C) than the untreated cellulosic material. This was presumably due to the attaching of sulfate groups on fiber surface during the hydrolysis step, which clearly reduced the activation energy for the degradation of nanocellulose product, making the nanomaterial less resistant to pyrolysis. Additionally, the lower degradation temperature of CNC_H2SO4_ could be due to the smaller fiber dimension, resulting in more surface areas exposed to the heat source and the partial disruptions of crystal structure compared to that of cellulose precursors [22]. These findings are in good accordance with Kallel’s study [56], in which the nanocellulose isolated from garlic straw by H_2_SO_4_ hydrolysis started to degrade at a lower decomposition temperature (200 °C) than that of its starting material (220 °C). In summary, the introduction of the sulfated groups and massive decrement of molecular weight significantly decreased the thermal stability of CNC.

Compared to CNC_H2SO4_ prepared via H_2_SO_4_ hydrolysis procedure (T_max_ = 273 °C), the CNC_Cr(NO3)3_ produced using Cr(NO_3_)_3_ hydrolysis system rendered much higher thermal stability (T_max_ = 344 °C) than its raw material (T_max_ = 295 °C), which can be explained by the fact that less damage happened in crystalline regions of the cellulose matrix benefiting from the milder hydrolysis conditions. In addition, the additional thermal stability is affected by the crystalline structure of cellulose, which increased as a result of the Cr(NO_3_)_3_ hydrolysis treatments. Some studies [57] reported that the high crystallinity nanocellulose could lead to higher thermal stability, but the thermal stability of high crystallinity sulfated CNC was reduced. Similar results have been reported by Mohamed et al. [42] where the nanocellulose derived from newspaper pulp rendered a lower T_max_ value (187.4 °C) than its starting material (346.4 °C) although it had higher crystallinity (90.15%) compared to untreated pulp (82%). In fact, the authors believed that the thermal destruction of cellulose was primarily affected by the presence of sulfate groups from H_2_SO_4_, in which the lower thermal stability was correlated to the content of sulfur impurities as well as removal of thermostable minerals during acid hydrolysis of the MCC. 

As pointed above, sulfuric acid hydrolysis leads to reduced thermal stability of acid hydrolyzed CNC compared to that of neat cellulosic feedstock. However, this effect can be diminished by neutralizing the CNC_H2SO4_ with NaOH solution (~1% NaOH). After neutralization with NaOH, no acid sulfate groups remained, and the thermal stability of neutralized CNC_Cr(NO3)3_ was shifted to a higher temperature. However, the presently used method for isolating the CNC_Cr(NO3)3_ has some restrictions, such as prolonged production time and capital cost in order to remove the free sulfuric acid remaining on the CNC_Cr(NO3)3_ surface after hydrolysis process, for their use in industrial scale. Thus far, the residual sulfuric acid in the cellulosic fiber is usually eliminated by the dialysis process against water until neutral pH is achieved, which is a costly procedure and usually takes a long time (3–7 days) [58]. Some studies reported that the presence of the divalent group (SO_4_^2−^) or anionic (–OSO_2_O–) of HSO_4_ and sulfates on the surface of nanocellulose may significantly reduce the interaction of cellulose fibrils with other polymer and biopolymer materials [59]. Even if the chemical neutralization method is simpler with less processing steps to produce unsulfated CNC_H2SO4_ than time-consuming dialysis process, the large amount of waste effluents (salts) produced during the neutralization process needs to be further recycled before being discharged into the local waterway or ecological system. In fact, the sulfur content is only able to affect the thermal behavior of the nanocellulose, but maybe not influence or cause a negative effect on the crystallinity of the H_2_SO_4_ hydrolyzed CNC [58]. In this study, the thermal degradation pattern of neutralized CNC_H2SO4_ was similar as native cellulose and CNC_Cr(NO3)3_, in which the low-temperature degradation peak in the DTG curve disappeared completely after neutralization. The neutralized CNC_H2SO4_ started to degrade at the temperature of ca. 237 °C. As expected, the T_max_ value of neutralized CNC_H2SO4_ profoundly increased to 349 °C, which can be explained by two main reasons. First, the absence of amorphous regions in the treated cellulose matrix and the highly structured crystals segments rendered the higher-temperature degradation. Second, the replacement of hydrogen ions (H^+^) from acid hydrolysis by alkaline ion (Na^+^) inhibited the dehydration catalyzed by H_2_SO_4_ [58].

After 500 °C, the thermal deposition temperature of all the cellulosic samples leveled off, and a slow thermal degradation profile was attained. This can be accredited to the further carbonization of polysaccharide chains initiated by the cleavage of C–C and C–H bonds. According to Figure 6, it is interesting to note that the sulfated-CNC_H2SO4_ possesses the highest residues (black char) at 600 °C as compared to that of other cellulosic sample. This behavior was attributed to that the sulfate groups in the CNC_H2SO4_ tend to play the role of flame retardant by promoting the dehydration reactions, which is conducive to the production of anhydrocellulose and leads to its decomposition to carbonaceous residues, as reported by other authors as Mohamed and his group [42]. Moreover, the CNC_H2SO4_ product with the acid sulfate group might be decomposed by more pathways by the action of acid, and, eventually, the intermediate products obtained, which could be further decomposed into the char residue, were more complex compared to the cellulose with acid content. Therefore, it is expected that char yield of neutralized CNC_H2SO4_ (6.57%) is remarkably lower than sulfated CNC_H2SO4_ (17.4%), considering the lack of remnant surface sulfate in the CNC_H2SO4_. It is quite interesting to note that the char level of neutralized CNC_H2SO4_ and CNC_Cr(NO3)3_ (6.57%–9.03%) was much lower than native cellulose (10.2%). The decreased char residue for the nanocellulose sample may be mostly related to the increased split hydrogen bonds and/or due to the absence of non-cellulosic components in the treated nanocellulose after hydrolysis treatment [60]. Furthermore, the presence of alkaline ions with highly polar field might act as the catalyst for complete degradation of glycosidic linkages via the hemolytic mechanism, which resulted in the decrement of char yield [61].

In view of the above thermogravimetric analysis, it was concluded that the produced CNC_Cr(NO3)3_ product from cellulosic feedstock exhibits better thermal stability than sulfated CNC_H2SO4_, and it will be more suitable for the production of green bio-nanocomposites as the typical processing temperature for polymer synthesis (i.e., thermoplastics) is normally higher than 200 °C. In this sense, CNC_Cr(NO3)3_ with more thermal stability than that of MCC and sulfated CNC_H2SO4_ provide better-reinforcing capability for different applications such as cosmetics, thermal sensitive papers, and disposable products, owing to the high elastic modulus of its crystal domains [51]. Table 2 summarizes the values of T_max_ for cellulose and its nanomaterials derived from different species and collected from the literature. 

### 2.10. Visual Appearance of Nanocellulose

**Figure 7:**
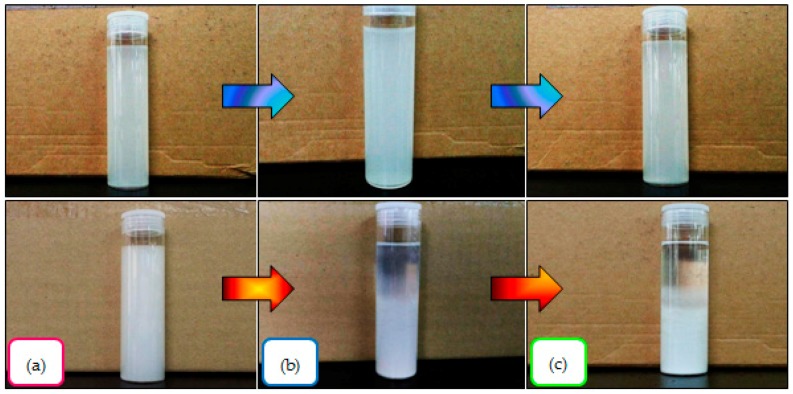
The electronic photos of the CNC_H2SO4_ (**top**) and CNC_Cr(NO3)3_ (**bottom**) suspension obtained at different period: (**a**) Right after sonication treatment; (**b**) After 24 h; and (**c**) After 7 days.

Figure 7 shows photographs illustrating the visual appearance of the aqueous suspension of CNC_H2SO4_ and CNC_Cr(NO3)3_ samples with a white light source. For comparison, all nanocellulose samples were dispersed in water at the same concentration. After the ultrasonication process, the electronic photos of CNC_H2SO4_ and CNC_Cr(NO3)3_ suspensions in the sample bottle are taken at 0 h, 24 h and 168 h. As can be seen in Figure 7a, two milk-like turbid suspensions were produced, suggesting that CNC_H2SO4_ and CNC_Cr(NO3)3_ both formed stable dispersions in water, with the latter being whiter than the former. After 24 h standing, the turbid phenomena continued for CNC_H2SO4_ colloidal suspensions, as shown in Figure 7b. As the time prolonged, however, some sedimentation from CNC_Cr(NO3)3_ were flocculated, whereas the good stability of CNC_H2SO4_ suspension could be kept at ambient temperature condition. For CNC_Cr(NO3)3_, the original suspension had separated into a two-layer mixture, which consists of a clear layer and a turbid layer that can be visibly observed. This could be attributed to the fact that no surface groups have been attached to CNC_Cr(NO3)3_ when prepared from Cr(NO_3_)_3_ metal salt catalyst, and thus the colloidal dispersion was expected to be less stable than CNC_H2SO4_ hydrolyzed by sulfuric acid. The high stability of sulfated CNC_H2SO4_ suspension can be achieved due to the sulfate negative charges (SO_4_^2−^) on the CNC_H2SO4_ surface being able to create a strong electrostatic repulsion between the cellulose surfaces, and these charged groups limit their ability to flocculate [24]. For this reason, CNC_Cr(NO3)3_ showed a higher risk for agglomerate in a water medium.

Due to the unmodified surface of CNC_Cr(NO3)3_ with the high aspect ratio and high content of hydroxyl groups on the fiber surface, the CNC_Cr(NO3)3_ tends to intertwine with their neighbor fibers and these networks were caused by the physical entanglement lock between the fibers. Furthermore, the lack of imparting electrostatic stability on CNC_Cr(NO3)3_ resulted in poor stability, leading to agglomeration as compared to the sulfated CNC_H2SO4_ which remained individually dispersed. A similar observation has been reported by others [49,64]. As a result, the CNC_Cr(NO3)3_ suspension was unable to maintain its stability after being stored for seven days and obvious precipitation can be seen, while the CNC_H2SO4_ still remained a milky colloidal suspension with negligible precipitation, as displayed in Figure 7c. In summary, the excellent stability of CNC_H2SO4_ suspensions was beneficial to reinforce the hydrophilic polymers (such as polyvinyl alcohol). Further studies on the potential reinforcement of composites by the CNC_H2SO4_ and CNC_Cr(NO3)3_ manufactured in this study are underway. 

### 2.11. Comparison between H_2_SO_4_ and Cr(NO_3_)_3_ Hydrolysis System

When comparing the two nanocellulose production procedures, the important differences are noticed in terms of environmental impact, productivity, fiber quality, and economic point of view. For CNC_H2SO4_ production, concentrated sulfuric acid (64 wt %) is required; however, the strong acidic waste effluent is a potentially harmful hazard to the biological system or nearby environment if the acidic wastewater is being discharged without any purification treatment. Therefore, it is a necessary step to neutralize this acid waste with base chemicals (i.e., KOH, NaOH, or NaHCO_3_) in the waste neutralization system before it can be safely released into the environment. In contrast, the Cr^3+^-containing solution produced from the Cr(NO_3_)_3_ catalytic system can be treated using several effective methods [65]. Among these techniques, chemical precipitation is worth mentioning as it is the most effective way for converting the Cr^3+^ to Cr(OH)_3_ via precipitation process with the presence of aqueous ammonia or sodium hydroxide. For commercial use, Cr(OH)_3_ is widely used as a pigment, mordant, and catalyst for organic reactions. This means that Cr(NO_3_)_3_ catalyst can not only produce value-added nanocellulose (solid residue) from the low-cost cellulosic feedstock, the generated supernatant may also be transferred into another useful byproduct, rather than discharged into the wastewater treatment plant without any profit. Approximately 9 kg of H_2_SO_4_ is required to produce 1 kg of CNC_H2SO4_ and the industry facing the difficulties in acid recovery economically. Therefore, the Cr(NO_3_)_3_ hydrolysis system could be sustainable and economically feasible. This will be the objective of further studies.

Additionally, the CNC_Cr(NO3)3_ produced via Cr(NO_3_)_3_ system rendered a better thermal stability than that of sulfated CNC_H2SO4_. This could make the CNC_Cr(NO3)3_ a promising candidate for reinforcing agent and gas-barrier material, as well as providing convenience for polymer applications and processing performed under high temperatures, especially thermoplastics. Oppositely, the introduction of ester groups on the surface CNC_H2SO4_ could enhance its stability in suspension, which is beneficial for the manufacture of nanocomposites. Unfortunately, the uncontrollable degradation of cellulose into oligomers liquid fraction is a common phenomenon observed from H_2_SO_4_ hydrolysis, resulting in a very low yield (*~*55%). In other words, extra attention is necessary during the process not only due to the highly corrosive acid treatment, most importantly to prevent the acid hydrolysis process going too far. Table 3 displays an exhaustive list of nanocellulose obtained from numerous lignocellulosic biomasses by different hydrolysis techniques, detailing the reaction conditions and presenting the physicochemical properties.

## 3. Materials and Methods

### 3.1. Materials

Microcrystalline cellulose (MCC) was purchased from Sigma-Aldrich (Saint Louis, MO, USA). The chemical compositions of the native cellulose determined according to the ASTM/TAPPI standard protocols are shown in Table 4. The hydrolyzing catalysts used in this study, namely sulfuric acid (H_2_SO_4_, 95%–97% purity) and chromium (III) nitrate (Cr(NO_3_)_3_), were bought from Merck (Kuala Lumpur, Malaysia). All chemicals were of analytical grade and used as received without further purification.

### 3.2. Preparation of CNC_Cr(NO3)3_ via Cr(NO_3_)_3_ Catalyzed Hydrolysis

In order to optimize the operating hydrolysis conditions, one-variable-at-a-time (OVAT) method was applied in this study to investigate the significance of every hydrolysis parameters in nanocellulose production. The catalytic hydrolysis reaction was initiated by reacting the native cellulosic material (1.0 g, oven dry weight) with Cr(NO_3_)_3_ solution (0.1–1.2 M) in a round-bottom flask with constant mechanical agitation using a heating mantle. The reaction was conducted at different temperatures (20–100 °C) and for different hydrolysis times (0.5–2.5 h). The solid–liquid ratio varied from 1:10 to 1:50. After the course of the reaction, the solid residue was separated from the reaction suspension via high-speed centrifugation (higher centrifuge speed can be used to improve separation) and washed thoroughly with cold deionized water until the wash water was maintained at constant pH. Afterward, the collected solid residue was subjected to lyophilization process and finally the fluffy white fiber product was obtained. Three replications were applied in each group of experiments in order to confirm the good reproducibility. The yield of CNC_Cr(NO3)3_ was determined by gravimetric analysis, as described elsewhere [9]. 

### 3.3. Isolation of CNC_H2SO4_ via H_2_SO_4_ Catalyzed Hydrolysis

The procedure for isolating of CNC_H2SO4_ was adapted from [70] with slight modification. Briefly, 1 g of cellulose material was dispersed in 100 mL deionized water through continuous mechanical stirring before the dropwise addition of 60 mL concentrated H_2_SO_4_ under an ice bath. This was to minimize a sudden temperature spike due to the exothermic reaction caused by the ion interaction between water and sulfuric acid. The suspension then heating up to 45 °C for 135 min. The reaction was terminated by diluting it with 10-fold cold water to avoid crystallization (iced water can be used to minimize dilution water usage while effectively terminating the hydrolysis reaction). The diluted suspension was centrifuged repeatedly to get the precipitate while removed the excess acid simultaneously by washing with deionized water. The supernatant was decanted off; fresh deionized water was added to the solids and mixed it well. This washing and centrifuging procedure were repeated until the supernatant was turbid, suggesting that CNC_H2SO4_ were being dispersed in solution. The turbid supernatant and settled solids were then mixed together and this slurry dialyzed against distilled water for 3–4 days until a constant pH was achieved, and freeze-dried to yield a white sulfate ester nanocellulose product. The dried product was stored in a vacuum for further product characterization.

### 3.4. Products Characterization Analysis

#### 3.4.1. Chemical Structure: Fourier-Transformed Infrared Spectral Analysis

Fourier transform-infrared spectroscopy (FTIR) of native cellulose and yielded nanocellulose were analyzed using a FTIR spectrometer (Bruker IFX 66/S, (PerkinElmer, Germany)) in order to determine the functional groups of the cellulosic product. In fact, the effects of the hydrolysis treatment on the chemical composition changes can be tracked from the FTIR spectra. The FTIR analysis was carried out in the transmittance mode with a wavelength range of 4000–400 cm^−1^ with a resolution of 4 cm^∓1^ at 32 scans. The samples were ground and mixed with KBr powder with a ratio of 1:100 (*w*/*w*), then pressed into an ultra-thin pellet before the analysis.

#### 3.4.2. Crystal Structure: X-ray Diffraction Studies

The impacts of the hydrolysis treatments on the crystallinity index of treated products can be investigated from the X-ray diffractograms. The crystallinities of native cellulose and isolated nanocellulose products were characterized by X-ray diffraction (XRD) study. The XRD patterns of the specimens were obtained from an X-ray diffractometer (PANalytical Empyrean) with a Cu-Kα radiation source which operated at 40 mA and 40 kV. The diffractograms were recorded from 5° to 60°. Crystallinity index (CrI) of samples was calculated by the peak deconvolution method as described by [71]. Generally, the crystallinity of samples was calculated by dividing the total peak area of all the crystalline peaks by the total peak of all non-crystalline plus crystalline peaks according to the Equation (5):

(5) $$\mathrm{CrI}\;(\%)\frac{A_{c}}{A_{c}+A_{n}}\times 100$$

where A_c_ and A_m_ are the area under the crystalline and non-crystalline regions, respectively.

#### 3.4.3. Morphological Analysis: FESEM and TEM Analysis

The surface morphology analysis of cellulose and nanocellulose products was studied with a field emission scanning electron microscope (FESEM; Hitachi SU8030, (Hitachi HTA, Schaumburg, IL, USA)) at an accelerating voltage of 5 kV. The samples were mounted on double-sided adhesive carbon tapes that stick on aluminum stubs, and coated with platinum using an auto-fine coater (JFC-1600, (JEOL, Ltd., Tokyo, Japan)) to improve conductivity and avoid over-charging. The elemental analysis of each sample was determined using EDX coupled with FESEM unit. The size and dimensions of yielded nanocellulose were further investigated by a transmission electron microscope (TEM; Tecnai G2 F20 Series, (FEI company, Hillsboro, OR, USA)) performed at a 200 kV acceleration voltage. To perform the TEM analysis, a nanocellulose suspension was treated with ultrasound for 3 min to separate agglomerated fibers. A droplet of the diluted suspension was deposited on a copper grid coated with a thin carbon film, and dried in a vacuum desiccator prior to analysis to ensure the samples were completely dry. The morphology and diameter of the yielded nanocellulose fibers were determined by analyzing the micrographs with ImageJ software (National Institutes of Health, New York, NY, USA).

#### 3.4.4. Thermal Property Investigation: Thermogravimetric Analysis (TGA) Study 

The thermal stability of samples was determined by a thermogravimetric analyzer (Model Q500 brand TA) under a nitrogen atmosphere. The sample was put in an aluminum cup and heated from 25 to 600 °C at a constant heating rate of 10 °C·min^−1^ in order to prevent any thermo-oxidative degradation.

#### 3.4.5. Visual Examination

The changes in dispersion of the CNC_H2SO4_ and CNC_Cr(NO3)3_ suspension after the hydrolysis process were studied through visual appearance in the aqueous medium (water). For this purpose, each nanocellulose aqueous suspension was prepared with a ratio of 2 mg/mL and the cellulose–water slurries were simultaneously treated by ultrasonication for 10 min in order to disperse CNC_H2SO4_ and CNC_Cr(NO3)3_ thoroughly. The pictures of samples were taken immediately after the ultrasonic treatment and then allowed to stand motionlessly for 1 day and 7 days at room temperature and atmospheric pressure before further photographing. 

## 4. Conclusions

A comparative study on two types of nanocellulose products, namely CNC_H2SO4_ and CNC_Cr(NO3)3_, derived from native MCC feedstock using concentrated H_2_SO_4_ and Cr(NO_3_)_3_ hydrolysis system, respectively, was conducted in this work. The optimal conditions for production of CNC_Cr(NO3)3_ given by RSM methodology were 80 °C, 1.5 h, 0.8 M Cr(NO_3_)_3_ catalyst and solid–liquid ratio of 1:30. Under these experimental conditions, the manufactured CNC_Cr(NO3)3_ displayed a spider-web-like fibrous morphology with a higher crystallinity of 86.5% ± 0.3%, yield of 83.6% ± 0.6%, and T_max_ value of 344 °C. For comparison, the CNC_H2SO4_ hydrolyzed by H_2_SO_4_ exhibited a rice-shaped structure with the crystallinity index of 81.4% ± 0.1%, yield of 54.7% ± 0.3%, and T_max_ value of 273 °C. Due to their crystallinity and aspect ratio differences, the isolated CNC_Cr(NO3)3_ with higher crystallinity, higher aspect ratio (about 15.7) and more thermal stability could potentially be used as reinforcement material for application in various fields targeting specific properties. In conclusion, the proposed Cr(NO_3_)_3_ hydrolytic system is easy to implement and permits producing more thermally-stable nanocellulose with high yield under milder hydrolysis conditions, thus eluding the customary usage of conventional concentrated H_2_SO_4_ treatment for producing better quality nanocellulose.

## Figures and Tables

**Table 1:** Elemental chemical analysis and atomic fraction of CNC_H2SO4_ and CNC_Cr(NO3)3_.

Element	CNC_H2SO4_	CNC_Cr(NO3)3_
	Mass Fraction (%)	Mass Fraction (%)
C	45.46	48.12
O	54.04	51.88
S	0.50	n/d

n/d: Not detected.

**Table 2:** Thermal profile of cellulosic material and its nanostructured product derived from various raw materials via different hydrolysis method in previously published studies.

Raw Materials	T_max_ Value (°C)		Hydrolysis Method	Reference
	Cellulose	Nanomaterial		
MCC	295	344	0.8 M Cr(NO_3_)_3_	This study
	295	210, 273	64 wt % H_2_SO_4_	
*Cannabis sativa*	315	265, 327	6 M H_2_SO_4_	[2]
Bamboo pulp	338	348	Ball milling + phosphotungstic acid	[53]
Bleached corncob residue	320	360	0.5% HCl + 88% formic acid	[22]
	320	336	PFI refining	
Industrial sludge	330	335	Ultra-fine grinding	[62]
Wood (Needle fir)	331	333	Ultrasonication	[63]

**Table 3:** Summary of the most important characteristics (i.e., hydrolysis conditions, crystallinity index, and fiber morphology) of produced nanocellulose derived from different biomass.

Source	Hydrolysis System	Width (nm) ^a^	Length (nm) ^a^	CrI (%) *	Yield (%)	Reference
MCC	0.8 M Cr(NO_3_)_3_	29.1 ± 7.8	455.73 ± 38.1	86.5 ± 0.3 **	83.6 ± 0.6	This study
	64 wt % H_2_SO_4_	9.9 ± 3.2	34.8 ± 14.2	81.4 ± 0.1 **	54.7 ± 0.3	
Hardwood pulp	Phosphotungstic acid	15–25	600–800 nm	85	60	[20]
Flax stem	Ultrasonication treatment	15–100	ns	81.6	ns	[63]
Wood (*Abies nephrolepis*)		10–20		71.0		
Moso Bamboo		10–40		64.9		
Wheat straw stem		15–35		63.4		
MCC	Ultrasonic-assisted enzymatic hydrolysis	<10	40–50	82.26 ± 0.11	22.57	[66]
Eucalyptus pulp	H_2_SO_4_ hydrolysis	3.6	ns	81	ns	[50]
	Multi-pass high pressure grinding	20		64.4		
Bamboo pulp	Ball milling + phosphotungstic acid	25∓50	200∓300	79.6	88.4	[53]
Groundnut shells	65 wt % H_2_SO_4_	5‒18	67‒172	74 **	31.3	[67]
Lotus leaf stalks	High intensity ultrasonication	20 ± 5	Micron scale	70	ns	[35]
*Phyllostachys nidularia* Munro	64 wt % H_2_SO_4_ + low intensity ultrasonication	5‒20	ns	69.32	ns	[68]
Sugarcane bagasse	High-pressure homogenization	<10	ns	68.10 ± 0.18	ns	[32]
Eucalyptus wood pulp	64 wt % H_2_SO_4_	5‒10	100‒300	64.4	ns	[24]
	37 wt % HCl	Up to tens of nm	Several microns nm	61.8	ns	
*Bambusa rigida*	33 wt % H_2_SO_4_ + ultrasonication	10‒30	ns	61.21	ns	[41]
Old corrugated container	Phosphoric acid + enzymatic hydrolysis + sonication	15–80	100–400	57.8	23.98	[60]
Kraft eucalyptus pulp	Enzymatic hydrolysis + homogenization	20	500	57 ***	ns	[69]
Corncob residue	64 wt % H_2_SO_4_	5.5 ± 1.9	198 ± 51	55.9	34.5	[22]

^a^ Morphological dimensions of the nanocellulose product was determined based on TEM analysis; Remark: Crystallinity values between studies in general should be compared carefully because different measurement methods have been used. * Calculated by Segal’s empirical method; ** Determined by peak deconvolution method; *** Estimated by FT-Raman spectroscopic method; ns: not specified.

**Table 4:** Chemical compositions of the microcrystalline cellulose feedstock.

Component	Test Method	Percentage (%)
α-cellulose	ASTM D 1103-55T	93.6
Hemicellulose	ASTM D 1104-56	0.8
Lignin	ASTM D 1106-56	<0.3
Ash	ASTM D 5360	10.4
Moisture content	ASTM D 2495	2.3
Extractive	TAPPI T257 cm-02	n/d

n/d: Not detected.
